# Patient-derived xenograft model for uterine leiomyoma by sub-renal capsule grafting

**DOI:** 10.14440/jbm.2018.243

**Published:** 2018-04-16

**Authors:** Vanida Ann Serna, Takeshi Kurita

**Affiliations:** Department of Cancer Biology and Genetics, The Comprehensive Cancer Center, Ohio State University, Columbus, OH, USA

**Keywords:** estradiol, fibroids, myometrium, NSG mouse, patient-derived xenograft, preclinical model, progesterone

## Abstract

Uterine leiomyoma (UL) or fibroid is a benign smooth muscle tumor of the myometrium with a lifetime incidence of approximately 70%. ULs often require medical intervention due to severe symptoms such as heavy menstrual bleeding and abdominal pain. Although the most common and effective management of ULs is surgical removal, the invasive surgical procedure imposes physical and psychological burdens on the patients. Moreover, the economic burden of UL on health care system is enormous due to the high cost of surgeries. Thus, therapeutic options with long-term efficacy to replace surgical management are urgently needed. For the development of such medical options, reliable preclinical research models are imperative. *Ex vivo* culture of UL cells has been the primary research model for decades. However, recent studies demonstrated that primary cell culture is not a suitable model for UL research, as primary cultures of ULs mostly consist of non-tumor fibroblasts. Here we describe the protocol for patient-derived xenograft of UL, which faithfully replicates the phenotypes of human UL *in situ*.

## BACKGROUND

Uterine leiomyoma (UL) or fibroid is a benign smooth muscle tumor of the myometrium with estimated lifetime incidences of > 70% [1]. Approximately one-fourth to one-third of women bearing ULs requires intervention due to symptoms, which include heavy menstrual bleeding, pelvic pressure and pain. Currently, the most effective and common treatment for ULs is surgical removal. However, physiological, psychological and economic burdens of UL surgeries on patients and their families are enormous [2]. Moreover, the annual medical cost of ULs in the United States has been estimated 4.1–9.4 billion US dollars [3]. Therefore, it is imperative to elucidate the mechanisms underlying the pathogenesis of UL in order to develop effective non-surgical therapies.

The epidemiology of ULs parallels with ovarian activity: there have been no single case of UL reported in prepubertal girls; symptomatic ULs occur mostly during the ages of 30–40 years; the prevalence increases with age; and the symptoms diminish by the time of menopause with tumor volume reduction. However, the symptoms may continue in menopausal women receiving hormone replacement therapies, indicating that UL is dependent on ovarian steroid hormones. Between two ovarian steroids, 17β-estradiol (E2) and progesterone (P4), E2 was believed to be the primary growth promoter of ULs for decades based on experimental evidence from animal and cell culture models. Meanwhile, studies with human tumors *in situ* suggested that P4 played a more important role than E2 in the growth of ULs. The direct evidence for the mitogenic effect of P4 on ULs was first demonstrated by a study with this patient-derived xenograft (PDX) model [4], and the findings in the PDX study were consistent with outcomes from the clinical trials of Selective Progesterone Receptor Modulators (SPRMs) for ULs [5].

Recent comprehensive genomic analyses identified recurrent mutations associated with ULs, revealing the presence of at least 4 UL subtypes with unique genetic alterations [6,7]. The most common subtype is *MED12* mutant leiomyoma (MED12-LM), which accounts for approximately 70% of all UL cases [6-9]. MED12-LMs consist of a similar number of MED12-mutant smooth muscle cells (SMCs) and *MED12*-wild-type tumor-associated fibroblasts (TAFs) [10]. Accordingly, the primary culture of MED12-LMs is not suitable to study ULs, as MED12 mutant SMCs quickly disappear when TAFs overwhelm the culture [11,12]. In cancer research, PDX has become a standard research model to fill the gap between *in vitro* and clinical studies [13]. Here we describe the technical details of the subrenal capsule PDX optimized for ULs as an ideal alternative to cell cultures.

## MATERIALS

### Animals

In a systemic comparison of nude, SCID, NOD/SCID, and NOD/SCID *Il2rg* null (NSG) mice as hosts of UL PDXs, the best take rate was achieved with NOD/SCID and NSG hosts. Nude mice were unsuitable as hosts because they rejected LM PDXs [14]. In our laboratory, we use NSG mice (Jackson Laboratories 005557, Bar Harbor, ME), as the take rate of UL PDXs on the subrenal grafting site is nearly 100%. Since the method involves hormone treatments, we use young adult females at 8–12 weeks of age. All mice must be used in accordance with institutional and governmental requirements [4,15]. All animal procedures were performed in accordance with the Institutional Animal Care and Use Committee of The Ohio State University (Protocol # 2014A00000060) and Northwestern University (Protocol # 2013-1937).

### Human ULs

UL tissues should be immediately transferred into ice-cold DMEM/F12 medium upon extraction and transferred to the lab within 8 h. Studies involving humans must be approved by the institutional review board, and all participants must have given written consent to the study protocol in advance.

### Reagents

#### Surgery

✓ Ketamine hydrochloride (100 mg/ml) (Hospira, Lake Forest, IL, NDC 0409-2051-05)
✓ Xylazine (20 mg/ml) (Akorn Animal Health, Lake Forest, IL, NADA 139-236)
✓ Buprenorphine (0.3 mg/ml) (Reckitt Benckiser, Hull, England, NDC 12496-0757-1)
✓ Meloxicam (Eloxiject) (5 mg/ml) (Henry Schein, Dublin, OH, NDC 11695-6925-2)
✓ Decon CiDehol 70% Isopropyl alcohol, 16 oz spray bottles (ThermoFisher Scientific [TFS], Waltham, MA, 04-355-63)
✓ Surgical Scrub and Handwash 2% Chloroxylenol (Vetoquinol, Ft. Worth, TX, 1703000826) or 10% Povidone iodine prep pads (TFS 06-669-70)

#### Hormone pellet

✓ 17β-Estradiol (Millipore-Sigma [Sigma], St. Louis, MO, E2758)
✓ Progesterone (Sigma P0130)
✓ Cholesterol (Sigma C3045)
✓ Diethyl Ether (Sigma 309958)

#### Isolation of UL cells and cell pellet preparation

✓ FBS (TFS, 16000044)
✓ DMEM/F-12 (TFS 11320082)
✓ Hanks’s Balanced Salt solution (TFS 14175095)
✓ Collagenase Type I (Sigma C0130)
✓ DNase I (Sigma D5025)
✓ Gibco Antibiotic-Antimycotic (100×) (TFS 15240096)
✓ 0.25% Trypsin-EDTA (TFS 25200114)
✓ Collagen I High Concentration, Rat Tail (Corning, New York, NY, 354249)
✓ Cell culture grade water
✓ 1N NaOH
✓ 10× PBS

### Recipes

#### Cell isolation cell pellet preparation

✓ Culture medium: DMEM/F-12 containing 10% FBS and Gibco Antibiotic-Antimycotic
✓ Tissue digestion medium: Hanks’s Balanced Salt solution containing 1.5 mg/ml collagenase Type I supplemented and Gibco Antibiotic-Antimycotic
✓ Collagen stock solution: Dilute collagen I to 3–5 mg/ml with sterile water. Snap-freeze aliquots (1 ml/tube) in liquid nitrogen, and store at –80°C. Thaw overnight at 4°C prior to use.
✓ Collagen setting solution: Mix 100 µl 10× PBS, 23 µl 1N NaOH and 877 µl cell culture grade water. Sterilize with a 0.2 µm syringe filter, and store at 4°C in an airtight small-void-volume container.

#### Reagents for mouse surgeries

✓ Ketamine/Xylazine Anesthetic (dosage 90/8 mg/kg ketamine/xylazine): Mix 0.9 ml Ketamine (100 mg/ml) and 0.14 ml xylazine (20 mg/ml), and dilute it to 10 ml with saline. Inject 100 µl per 10 grams body weight.
✓ Buprenorphine (dosage 0.1 mg/kg): Dilute 300 µl Buprenorphine (0.3 mg/ml) to 9 ml with saline. Inject 100 µl per 10 grams body weight.
✓ Meloxicam (dosage 2.0 mg/kg): Dilute 800 µl Meloxicam (5 mg/ml) to 10 ml with saline. Inject 100 µl per 20 grams body weight.
✓ Hormone pellets: Because murine hormone levels are far below that of humans, the host must be supplemented with both E2 and P4 to achieve the robust growth of xenografts. Dissolve cholesterol, progesterone (P4) and/or estradiol (E2) in ethyl ether in the following ratio by weight, 1% estradiol and/or 5% progesterone with cholesterol to 100 %. Evaporate solution overnight with stirring under the fume hood. Compress powder into 3 mm diameter pellets with the Parr 2811 Pellet Press. Cut pellets to ~30 mg (~2 mm length). Subcutaneous implantation of two 30 mg E2 + P4 pellets sustains serum E2 and P4 levels of 374.5 pg/ml (*N* = 20, 95% CI 291.5 to 457.5) and 50 ng/ml (*N* = 20, 95% CI 37 to 63), respectively, for 2 months. These are comparable to the serum hormone levels of cycling women [16]. Pellets should be replaced every 2 months.

### Equipment

✓ Disposable Scalpel #22 (World Precision Instruments, Sarasota, FL, 500354)
✓ Surgical instruments (Sterilize prior to procedure): Moria MC32/B Iris forceps (Fine Science Tools, Foster City, CA (FST), 11373-22), needle holder with suture cutter (FST 12002-14), scissor (FST 14054-13), hardened fine scissor (FST14090-09), student Vanna spring scissor (FST 91500-09), forceps (FST 11064-07), extra-fine Graefe forceps (FST 11150-10)
✓ Glass rod with balled tip (< 1 mm^3^ ball) made from 9 inch Pasteur pipets. Flame pipets ~20 mm below the tip, pull the tip and further burn the closed end to make a ball [17].
✓ Supplementary surgical tools: small animal clipper, cautery kit (FST 18010-00), wound clip applier, remover, and clips (Fisher 01-804, 01-804-15, 01-804-5)
✓ Coated Vicryl Suture 4-0 27'' (Ethicon, Cincinnati, OH, J310H)
✓ Exel International Tuberculin Syringes (FST 14-840-50)
✓ Dissecting microscope with LED illumination (*e.g.*, Leica, DMS1000, Buffalo Grove, IL)
✓ MultiSample BioPulverizer or single BioPulverizers (BioSpec Products Inc, Bartlesville, OK, 59012MS or 59012N)
✓ The Parr 2811 Pellet Press (Parr Instrument Company, Moline, IL)
✓ Biological Safety Cabinet
✓ Fumehood
✓ Magnetic stirrer and stirrer bar
✓ 500 ml beaker
✓ 125 ml or 250 ml baffled flask

## PROCEDURE

**NOTE:** Although we expect this protocol to work for all subtypes, this protocol is optimized for MED12-LMs, as this subtype is the most common though the most difficult to make xenografts: MED12-LMs generally contain a smaller number of tumor cells compared to HMGA2-LMs, as the bulk of tumor is made of rigid ECM and often bear a highly necrotic and calcified center. Myometrial control tissues may be processed in parallel.

**NOTE:** Sterile/aseptic techniques must be used throughout. Procedure must be performed in a biological safety cabinet.

### Isolation of UL cells

**Figure 1.**
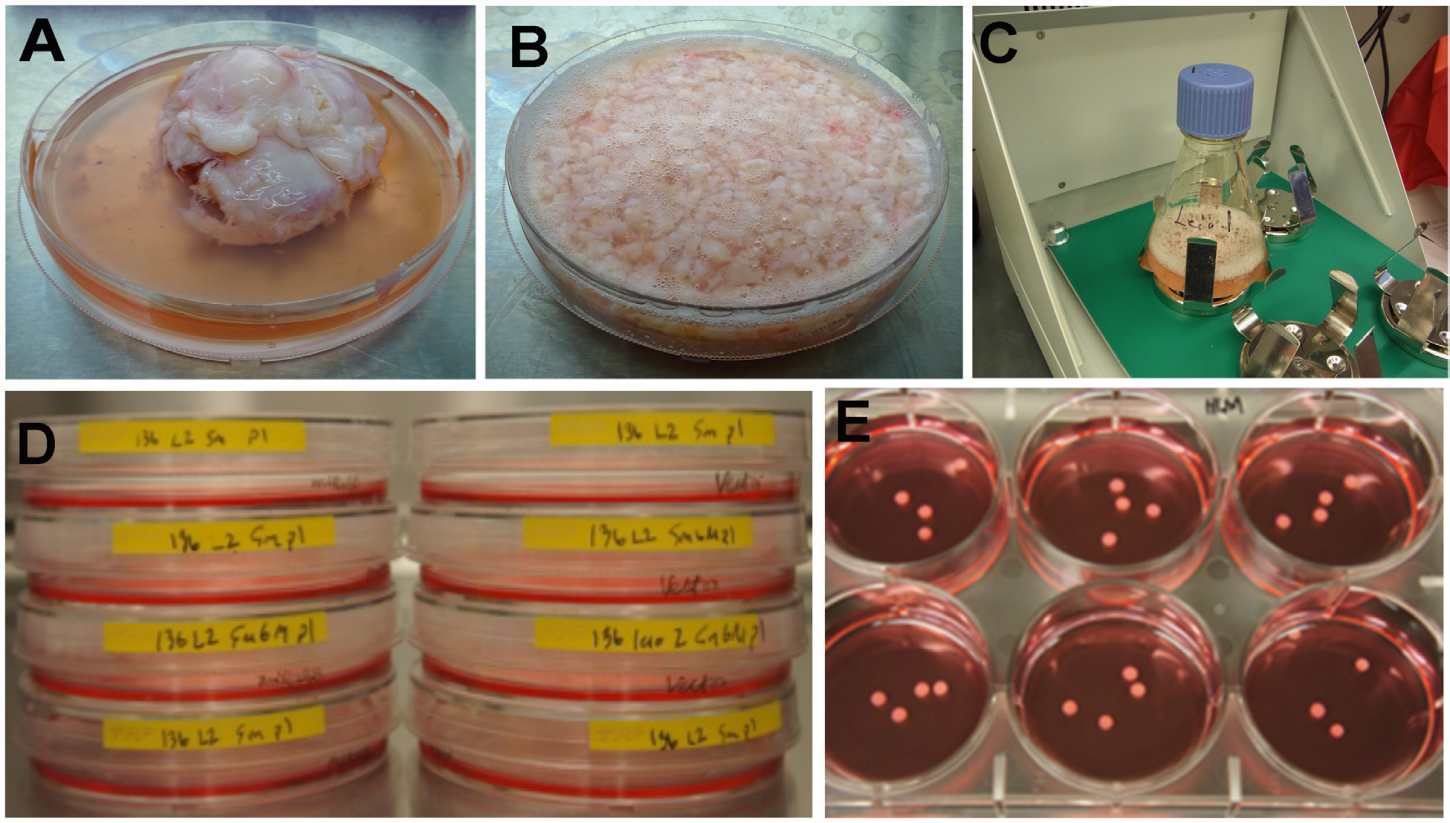
Tissue digestion, cell isolation and cell pellet preparation. **A.** Surgically removed UL. **B.** UL is minced in a 10 cm petri dish. **C.** UL tissue pieces are digested in a baffled flask that is set in a shaker-incubator. **D.** UL cells are plated in 10 cm culture dishes. **E.** Cell pellets are suspended in DMEM/F12.

1. Place UL tissues in cold DMEM/F-12 medium containing antibiotic-antimycotic soon after extraction (**Fig. 1A**).
2. Using a #22 surgical blade, cut the UL tissues into pieces of < ~9 mm^3^ in a 10 mm plastic petri dish, excluding any calcified or necrotic portions as well as myometrium. Keep tissue covered with tissue digestion medium (**Fig. 1B**).
  **NOTE:** If necessary due to time constraints, tissue pieces can be cut and stored in culture medium at 4°C overnight.
3. Suspend minced tissues into tissue digestion medium of a volume greater than five times tissue volume and transfer to 125 ml baffled flasks (< 50 ml) or 250 ml baffled flasks (< 80 ml) (**Fig. 1C**).
4. Add 10 µl DNase I stock per 10 ml tissue digestion medium.
5. Digest tissues at 37°C on a shaker 200–250 rpm until the pieces of tissue disappear. When digestion takes longer than 3 h, stop the shaker, wait for large tissue pieces settle on the bottom, and then transfer the digestion medium containing cells into 50 ml polypropylene tubes on ice. Add fresh digestion medium to the flask. Restart the shaker to digest tissues until large pieces disappear. Digestion time < 6 h is recommended for the best result.
6. Filter the cell suspension through a 100 µm Falcon cell strainer (BD Falcon) into new 50 ml polypropylene centrifuge tubes.
7. Centrifuge at 220× *g* for 5 min at 4°C.
8. Remove supernatant. Gently resuspend cells in 45 ml culture medium.
9. Centrifuge at 220× *g* for 5 min at 4°C again.
10. Remove supernatant. Gently resuspend cells in 10–20 ml of culture medium.
11. Count cells.
12. Plate cells on 10 cm culture dish at ~2–4 × 10^6^ cells per plate in the culture medium, and incubate for 1–3 d (**Fig. 1D**).
  **NOTE:** The singly isolated cells can be directly prepared into cell pellets. Nevertheless, UL cells are usually plated for a short period to removed dead cells that do not attach the plate. Longer incubation is not recommended, as it increases the concentration of TAFs.

### Cell pellet preparation

***13.*** Collect cells from culture dish.
  ***13.1.*** Aspirate culture medium. Gently wash cells with ~8 ml of pre-warmed PBS (Mg^++^ and Ca^++^ free) twice.
  ***13.2.*** Rinse the surface of cells with 1 ml of 0.25% EDTA-Trypsin, and aspirate.
  ***13.3.*** Add 2 ml of 0.25% EDTA-Trypsin, and incubate at 37°C until cells detach. If a majority of cells are still attached after 15 min, transfer the trypsin solution into a 15 ml centrifuge tube with 5 ml culture medium. Add 2 ml of fresh 0.25% EDTA-Trypsin solution to the culture dish. Incubate at 37°C for an additional 15 min.
  ***13.4.*** Dispense 5 ml of culture medium to the culture dish. Collect detached cells by pipetting several times.
  ***13.5.*** Transfer the cell suspension to a 15 ml centrifuge tube. Centrifuge at 220× *g* for 5 min.
  ***13.6.*** Remove the supernatant. Gently resuspend the cell pellet into ~5 ml culture medium.
  ***13.7.*** Count the cells in this suspension.
    **NOTE:** The cell number is sum of different cell types including SMCs, TAF and vascular cells (vascular smooth muscle, endothelial cells).
***14.*** Collect a volume of cell-suspension that contains the number of cells required to make the desired number of grafts. Transfer it into a centrifuge tube.
***15.*** Centrifuge at 220× *g* for 5 min. Remove the supernatant. Calculate the volume of the collagen gel solution required to prepare desired number of cell pellets.
  **CRITICAL STEP:** The ratio of cells to collagen is of great importance: The higher the concentration of cells embedded, the softer the cell pellet. We recommend a pellet with 0.25 to 0.5 × 10^6^ cells in 20 µl collagen solution.
***16.*** Prepare collagen gel solution: mix one volume of collagen stock solution with one volume of setting solution, all on ice.
  **NOTE:** Collagen gel polymerizes slowly even on ice. Therefore, use it within 2 h.
***17.*** Resuspend cells collected in step15 in collagen gel solution using a pipette with a wide-orifice tip.
***18.*** With a wide-orifice tip, dispense droplets of desired pellet volume onto the surface of a cell culture plate (6 well-plate is recommended).
***19.*** Incubate the plate containing the droplets at 37°C in a CO_2_ incubator for 15–30 min. After confirming the droplets have solidified, add pre-warmed (37°C) DMEM/F-12 to a depth of 0.5–1.0 cm (~6 ml per well for 6 well plate), and gently detach the cell pellet from the bottom with the tip of a P20 micropipette (**Fig. 1E**).
  **NOTE:** The cell pellets in the medium can be maintained at 37°C in a CO_2_ incubator over night and grafted on the next day.
***20.*** Transfer cell pellets to surgery suite for grafting.

### Ovariectomy and xenografting

**NOTE:** While inhalational anesthesia can be used, the mouse position would be constrained to the inhalation tubing position. Therefore, injectable anesthesia, which removes restraints to position, is preferred. As this procedure involves human cells, the grafting surgery must be performed in a biosafety cabinet. We use a Leica DMS1000 digital dissecting microscope, as it allows monitoring the surgery through the cabinet window.

**Figure 2.**
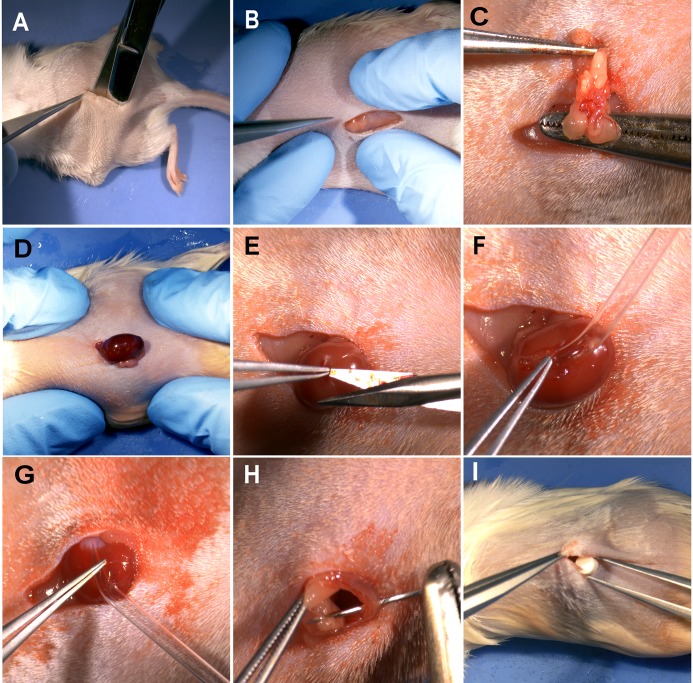
Ovariectomy, subrenal grafting and hormone pellet implantation. **A.** Skin is separated from the muscle wall. **B.** The ovarian fat pat is visualized to determine the location of the muscle incision. **C.** A hemostat is clamped below oviducts prior to removal of ovaries and oviduct. **D.** The kidney is exteriorized with gentle pressure to push it through the muscle incision. **E.** The capsule, held by the Moria iris forceps, is pierced by the spring scissors. **F.** The glass rod with ball-tip creates a pocket for the cell pellet between the capsule and kidney parenchyma. **G.** A cell pellet is pushed deep into the pocket by the glass rod. **H.** After returning the grafted kidney into the peritoneum, the muscle wall is sutured. **I.** A hormone pellet is inserted through the skin incision to the nape of the neck.

***21.*** Prepare clean post-surgical cage on a heating pad set to low.
***22.*** Intraperitoneally inject ketamine/xylazine anesthetic. When the mouse is unresponsive to foot pinching and whiskers unmoving, continue.
***23.*** Apply analgesics at this time as required by your institution.
***24.*** Apply eye lubricant, and shave dorsal caudal half of mouse.
***25.*** Treat the entire dorsal caudal half of the mouse with a disinfectant such as chloroxylenol, followed by 70% alcohol. Repeat thrice.
***26.*** Transfer mouse to a heating pad set to low.
***27.*** Make a 1.0 cm incision through the skin of the dorsal midline parallel to the spine. Separate the skin and muscle wall laterally by probing with scissors (**Fig. 2A**).
***28.*** Identify the ovarian fat pad through the muscle (**Fig. 2B**). Make a 2 mm incision perpendicular to the spine just rostral to the ovarian fat, avoiding blood vessels.
***29.*** Reach through both the skin and muscle incisions with forceps, grasp the ovarian fat, and pull the ovary through the incision to the exterior of the body. Hold the ovary away from the kidney, separating connective tissue if necessary.
***30.*** Remove the ovary by cauterizing arteries and connective tissues. Or clamp arteries and uterus just below the oviduct with a hemostat (**Fig. 2C**), then remove the ovary and oviduct with a fine scissors on the distal side of the hemostat. To avoid bleeding, wait for > 1 min before removing hemostat.
***31.*** Return the uterus into the peritoneal cavity.
***32.*** With gentle pressure from thumbs and forefingers positioned on the muscle wall, gently push the kidney out through the muscle wall incision (**Fig. 2D**).
  **NOTE:** The incision length is in opposition to the longitudinal axis of the kidney, such that the kidney is better maintained outside the muscle wall during the grafting surgery.
***33.*** Using the soft rounded tips of the Moria iris forceps, gently grip the kidney capsule and lift it away (1 mm) from the kidney parenchyma. Ideally the capsule is gripped at the outer edge of the kidney to optimize space for grafts. Pierce the capsule with one blade of the opened spring scissor (**Fig. 2E**), and move the blade into the capsule for the length of the blade. Make a single straight incision (~2 mm) along the longitudinal axis of the kidney.
***34.*** Dip the tip of the glass rod into DMEM/F12 in the dish of cell pellet. Holding one edge of the capsule at the incision with the Moria iris forceps, gently insert the wet tip of the glass rod through the incision between the capsule and kidney parenchyma to form a pocket (**Fig. 2F**). The pocket with a diameter of the pellet should extend far from the capsule incision but not to the hilus.
***35.*** While holding open the incision with the Moria iris forceps in one hand, gently pick up a single cell pellet from medium with forceps and insert it into the pocket.
***36.*** Use the glass rod to gently push the pellet deep into the pocket (**Fig. 2G**), and then release the capsule. To force the pellet deeper, lightly compress the capsule from the outside with the glass rod.
  **NOTE:** Additional pockets may be made from the same incision and pellets inserted on the same side of kidney. Multiple incisions in the capsule can be made in different locations, but care must be taken to not disrupt the overall integrity of the capsule.
***37.*** Holding the muscle incision open with forceps, gently guide the kidney back into the peritoneal cavity.
  **NOTE:** Do not push kidney with forceps tips, rather use the flat handle end or fingers.
***38.*** Close the muscle wall incision with absorbable sutures in the simple interrupted pattern (**Fig. 2H**).
***39.*** Repeat steps 28–38 on the contralateral side.
***40.*** Grasp the skin just rostral to the skin incision site with forceps. Probe apart the skin and muscle wall to form a tunnel from the incision site to the nape of the neck, using another forceps.
***41.*** With forceps holding the hormone pellet, push the pellet through the tunnel, and deposit the pellet at the nape of the neck (**Fig. 2I**).
***42.*** Release the skin and close the incision with wound clips or suture.
***43.*** Return the mouse to a clean cage on a heating pad. Once the mouse is ambulatory, monitoring can be discontinued and the cage returned to the rack. The mouse should be given analgesic in subsequent days and wound clips removed according to the approved IACUC protocol.

### Assessing tumor growth in live mice

**Figure 3.**
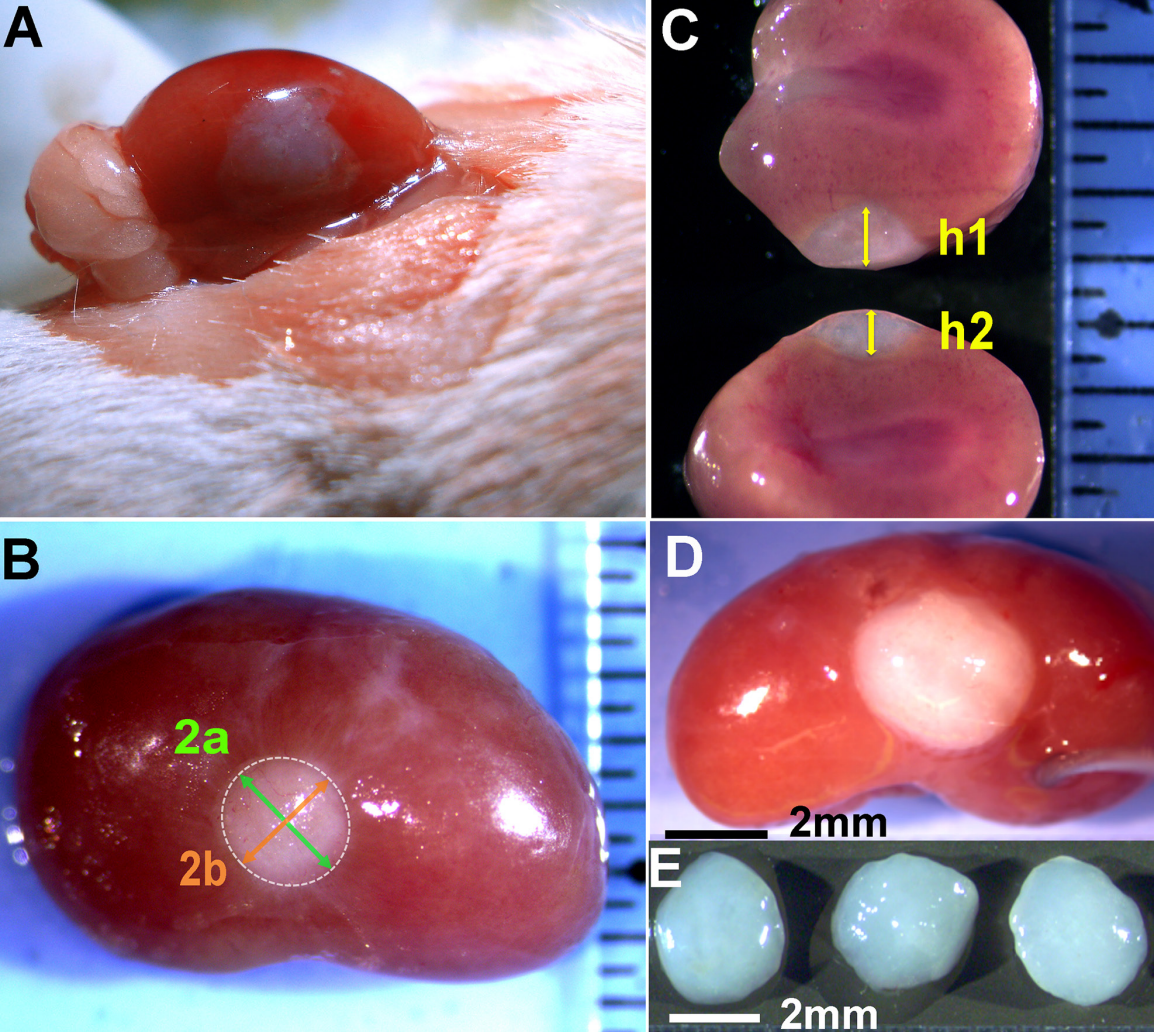
Assessing tumor growth. **A.** Assessing tumor growth in mice. Presented is a freshly grafted pellet. Thus, no growth is observed. **B** and **C.** Presented is a PDX after 8 weeks of growth in a host supplemented with E2 + P4. Dimensions of the PDX on the kidney are assessed for volume measurement. **D** and **E.** Isolation of xenografts for biochemical analysis. Grafts on the kidney (D) are collected free of kidney tissues (E).

  **NOTE:** Measurement of tumor volume by IVIS with transduction of reporter genes, such as RFP, is possible. However, the transduction and selection step significantly reduces the viability of SMCs, resulting in a TAF dominant culture, which in turn affects the growth rate of PDXs. Hence, we assess the growth at 4 weeks after transplantation by live surgery or necropsy, which is explained in the following section. Based on the size of PDXs at 4 weeks, the experimental schedule may be revised as needed.
  ***44.*** Follow steps 21–28, looking for the kidney rather than an ovarian fat pad. Visually check the growth of PDXs on the kidney through this incision, or expose the kidney following step 32 (**Fig. 3A**).
***45.*** Follow steps 37 and 38 if the kidney was exposed.
***46.*** Repeat on the contralateral side
***47.*** Then follow 42–43.

### Tissue collection

***48.*** Euthanize mouse. To obtain blood, anesthetize mouse with ketamine and xylazine. When full anesthetic depth is reached, perform cardiac puncture; or more preferably, obtain blood through enucleation, as there is less cell lysis.
***49.*** Spray mouse with 70% ethanol to minimize hair contamination. Cut through the skin and muscle walls, and expose the kidneys.
***50.*** Grasp the renal vessels at the hilus, and cut the kidneys away from the body.
***51.*** Transfer the kidney bearing PDXs to a small petri dish with PBS, still grasping by the vessels so as not to damage the kidneys.
***52.*** Image the grafts on the kidneys with a ruler in the viewer along the x-and y-axes (**Fig. 3B**). So long as the axes are perpendicular, one axis is set on the greatest diameter available.
***53.*** For an accurate assessment of height, cut through the center of the graft and kidney parenchyma to obtain a cross section which displays the entire height of the graft (**Fig. 3C**).
***54.*** Trim excess kidney tissues using a surgical knife, and process the PDX and surrounding kidney tissues for desirable histological analyses. For the extraction of nucleic acid and protein, remove kidney tissues completely, and freeze PDXs in liquid nitrogen.
  **NOTE:** Comparable to original human UL, MED12-LM PDXs are well-structured by rigid ECM (**Fig. 3D** and **3E**), making isolation of PDXs free of kidney tissues is relatively easy.
***55.*** Collect host female reproductive tract, and fix for future reference.

### Tumor volume measurement

***56.*** Calculate tumor volume (**Fig. 3B** and **3C**). Although the PDX grows as an ellipsoid as revealed upon complete extraction, the tumor volume has been assessed as a hemi-ellipsoid, the portion of tumor that extends above the surface of the kidney. Tumor volume = ⅔ πabc: a and b = radius on x and y axes, c = height = (h_1_ + h_2_)/4.

### Protein/RNA extraction method

  **NOTE:** Homogenization of MED12-LM PDX is particularly difficult due to rigid texture and small size. Additionally, because ECM accounts for a large portion of tissue mass, the yield of cellular protein and RNA is significantly low compared to other types of tumors. Accordingly, a frozen-crushing technique with the Bio-Spec Pulverizer is the preferred method for extraction of cellular materials. Pulverization of human-derived materials must be performed in a biosafety cabinet. Here we describe the method with an individual pulverizer.
  ***57.*** Place a clean BioPulverizer in a shallow container. Cool thoroughly with liquid nitrogen. Then place it on bench top.
***58.*** Place pre-frozen tissue in the well of the mortar, and insert pestle.
***59.*** Pound pestle with hammer multiple times to pulverize the tissue.
***60.*** Scrape the powder into a microcentrifuge tube with a sterile spatula.
***61.*** Clean the pulverizer between samples, and repeat as needed.
  **NOTE:** Should the pulverizer thaw, clean thoroughly, and repeat with fresh liquid nitrogen.
***62.*** Proceed with a standard protein and RNA extraction protocol.

## ANTICIPATED RESULTS

PDX prepared with isolated human uterine cells self-organize into tissues with comparable histology to the original tissues [4,18]. If the protocol is appropriately followed, one can expect nearly 100% of grafted UL cells to form ULs of measurable size as shown in **Figure** **3**. If undigested UL tissue transplants are used, the take rate will be significantly lower compared to this cell-pellet protocol, as thick layers of ECM in original UL tissue interfere with angiogenesis [4]. If UL cells are cultured for few days before preparation of cell pellets, the initial concentration of TAFs in MED12-LM PDX may exceed 90%. Nevertheless, E2 + P4 preferentially stimulates SMCs, and thus the tumor should form with an extended growth time.

The hormone condition described above is optimized to achieve fast tumor growth. Thus, the dosage of hormones should be modified according to the purposes of the study. Although PDX shrinks and tumor cells became dormant by hormone withdrawal, the survival of tumor cells in PDXs is hormone independent [4]. Thus, hosts may be left without hormone pellets for days after hormone pellet removal to clear the system.

**Figure 4.**
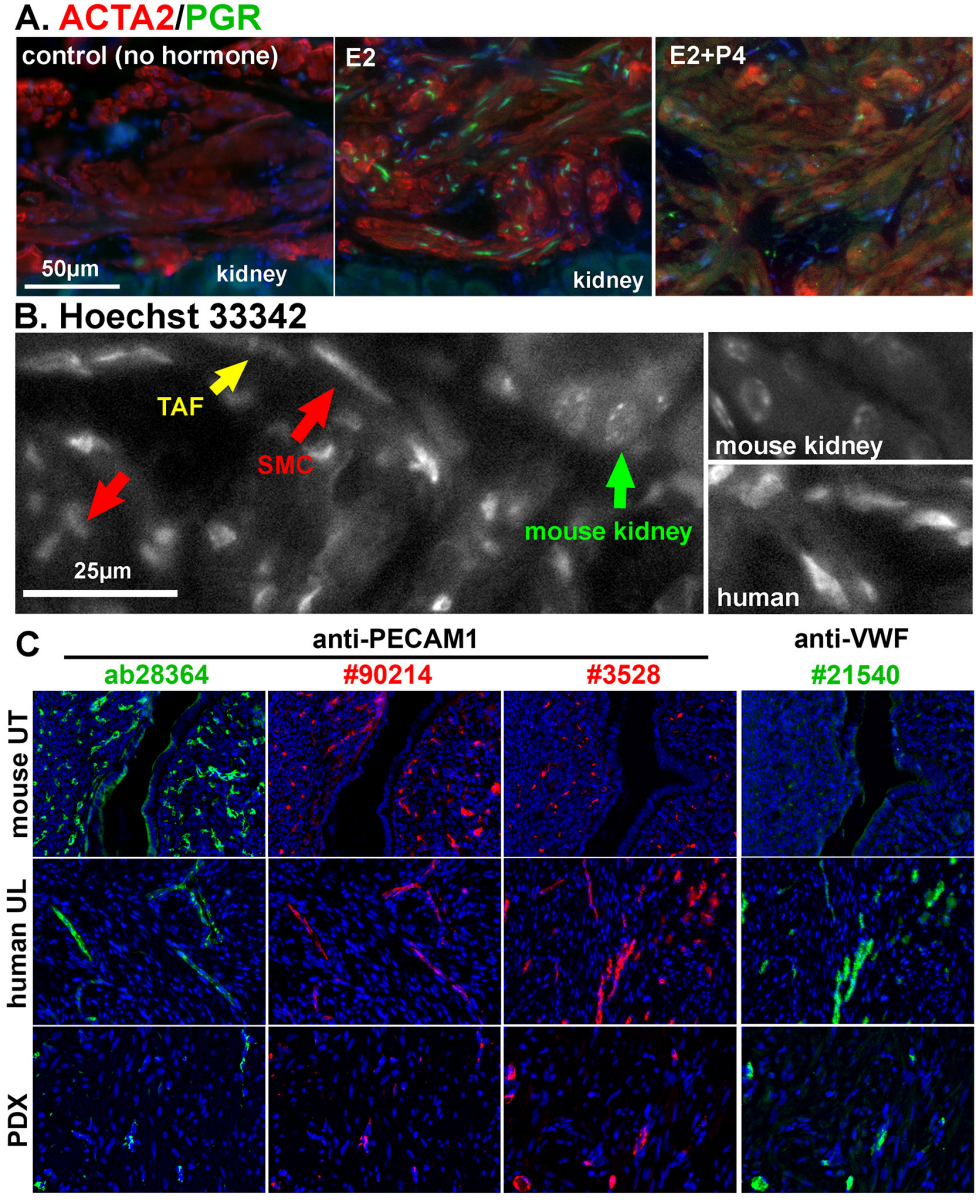
Histological analysis of PDXs. **A.** Double immunofluorescence analysis of ACTA2 (red) and PGR (green) in MED12-LM PDXs grown in hosts supplemented with no hormone (control), E2 and E2 + P4. Expression of PGR is E2 dependent. **B.** Identification of human and mouse cells by nuclear morphology. Human cells have diffuse nuclear staining; mouse cells have granular nuclear staining for heterochromatin. Also indicated are smooth muscle cells (SMC, red arrows) and tumor-associated fibroblasts (TAF, yellow arrows). **C.** Human origin of vascular endothelial cells in UL PDX. Two anti-PECAM1 (endothelial marker) antibodies, Abcam ab28364 (1:200) and Chemicon #90214 (1:200), preferentially stained mouse uterus (UT) over human ULs, whereas Cell Signaling Technologies #3528 anti-PECAM1 (1:100) antibody preferentially stained human endothelial cells. Chemicon #21540 anti-VWF antibody (1:200) was non-reactive with mouse endothelial cells. Almost all blood vessels in PDXs were strongly positive for VWF, indicating their human origin.

UL PDX response to E2 (and P4) can be detected by expression of progesterone receptor (PGR) (**Fig. 4A**) [4,10]. The technical details of immunostaining have been described previously [19]. Human and mouse cells can be distinguished by nuclear morphology [18,20]: Mouse nuclei are highlighted by bright granular staining for heterochromatins (**Fig.** **4B**). Furthermore, most cells in PDXs, including vascular cells, are of human origin (**Fig. 4C**). Human endothelial cells are often detected in the host kidney at the boundary with PDXs (not shown), indicating that LM cells have lower angiogenic activities than host kidney. Hence, a substantial concentration of vascular endothelial cells must be present in the original LM culture for the survival and growth of PDXs, given the contribution of host vascular cells to PDX is none to minimum.

## TROUBLESHOOTING

Possible problems and their troubleshooting solutions are listed in **Table 1**.

## Figures and Tables

**Table 1.** Troubleshooting table.

Step	Problems	Causes	Suggestions
5	Substantial tissue is undigested	The ratio of medium to the tissue is too small	Increase the volume of digestion medium
20	Pellet is too soft	The ratio of cell to volume of collagen solution is too high Check if setting solution neutralized the collagen solution	Reduce the cell concentration Increase the volume of setting solution or make new setting solution
32	Kidney slips back in	Incision on the body wall is too large	Attempt lithotomy, curving the body to maintain kidney outside the body
34	Capsule tears despite gentle handling	Capsule is too dry	Apply a drop of DMEM-F12 from pellet dish to kidney surface
49	Tumor didn’t grow in hosts with E2 + P4	Quality of original tumor was low (low SMC/TAF ratio) Tumor cell concentration in the PDX is too low as TAFs took over the culture used for cell pellet preparation Hormone pellet was lost	Check the concentration of SMCs in original tumor by IHC The particular set of PDXs is not suitable for experiments Prepare cell pellets using a new UL tissue Graft new hormone pellets if original pellets are not found and reassess experimental design
